# Effect of *Ishige okamurae* extract on musculoskeletal biomarkers in adults with relative sarcopenia: Study protocol for a randomized double-blind placebo-controlled trial

**DOI:** 10.3389/fnut.2022.1015351

**Published:** 2022-09-27

**Authors:** Sae Rom Lee, Ye Li Lee, Sang Yeoup Lee

**Affiliations:** ^1^Family Medicine Clinic and Biomedical Research Institute, Pusan National University Yangsan Hospital, Yangsan, South Korea; ^2^Integrated Research Institute for Natural Ingredients and Functional Foods, Yangsan, South Korea; ^3^Department of Medical Education, Pusan National University School of Medicine, Yangsan, South Korea

**Keywords:** dietary supplements, sarcopenia, *Ishige okamurae*, muscle, randomized clinical trial, adult, brown seaweeds, functional foods

## Abstract

**Introduction:**

Sarcopenia is a phenomenon in which skeletal muscle mass decreases with age, causing many health problems. Many studies have been conducted to improve sarcopenia nutritionally. *Ishige okamura* (IO) is a genus of brown algae and plays a role in anti-diabetes, anti-obesity, and myogenesis. However, the effect of IO extract (IOE) on human muscle strength and mass is unclear. Therefore, we will examine the impact and safety of consumption of IOE for 12 weeks on muscle strength and mass in middle-aged and old-aged adults with relatively low skeletal muscle mass.

**Materials and methods:**

A randomized controlled trial is conducted on 80 adults aged 50–80. A total of 80 participants will be enrolled in this study. Participants assign IOE-taking group (*n* = 40) and placebo taking group (*n* = 40). At a baseline and 12 weeks after treatment, the following parameters of the participants are checked: knee extension strength, handgrip strength, body composition, laboratory tests, dietary recall, physical activity, and EQ-5D-5L.

**Discussion:**

The present study will be the first randomized, double-blind placebo-controlled trial to examine the efficacy and tolerability of IOE supplementation in adults with relatively low muscle mass. The nutritional intake and physical activity that might influence muscle strength and mass will be considered as covariates for transparency of results. The results of this study will provide clinical evidence for sarcopenia patients with nutrient treatment.

**Clinical Trial Registration:**

www.clinicaltrials.gov/, Identifier: NCT04617951.

## Background

After reaching about 50% of the total body weight at a young age, the muscle mass decreases gradually after the age of 30, reaching nearly 25% by 75–80 (1). With age, reduced physical activity, decreased protein synthesis, decreased anabolic hormone concentration, and progressive loss of motoneurons lead to sarcopenia (2–4). Sarcopenia is a disorder of skeletal muscle that reduces muscle strength and size (5). Sarcopenia can rise the event of fragility, falls, hospitalization, and mortality (5–7). Therefore, sarcopenia has been recognized as a disease since its registration in the International Classification of Disease, Tenth Revision, Clinical Modification on October 1, 2016 (8, 9). In recent years, several pharmacological treatments, including synthetic drugs and hormones, have been tested for the improvement of sarcopenia in clinical trials (10–12). However, it has been reported that testosterone may increase the risk for cardiovascular adverse events. Selective androgen receptor modulators have been used to avoid the side effects of anabolic steroids, but constipation, dyspepsia, or nausea have also been observed in studies (10). Growth hormone increased lean body mass and decreased fat mass; however, adverse events such as soft tissue edema, arthralgias, and carpal tunnel syndrome were significantly increased in the clinical trial (11). Therefore, there has been growing interest in identifying functional food with few side effects to prevent sarcopenia. Functional foods that have been recently studied to be effective against sarcopenia include curcumin, resveratrol, catechin, soy protein, ginseng, and *Schisandra chinensis* Baillon extracts (12, 13). A previous study showed that sea grape (*Caulerpa racemosa*) extracts improved serum peroxisome proliferator-activated receptor γ coactivator 1α, which plays a role in promoting muscle tissue remodeling after 4 weeks in men with obesity (14).

*Ishige Okamura* (IO) is a genus of brown algae, growing attached to the rocks from Geoje Island on the southern coast to the southwest coast and all over Jeju Island, South Korea. It is also distributed and abundant throughout the coastal areas of many countries in the Northwest Pacific region (15). IO extracts (IOE) are known to have anti-obesity by inhibiting lipid accumulation and anti-diabetic effects by improving insulin resistance and protecting pancreatic β-cells dysfunction *in vitro and vivo* (16, 17). Lately, IO extract (IOE) has been reported to promote the pathway to synthesis of muscles through the myostatin/Smad3 pathway and enhance protein phosphorylation of the growth regulatory axis *in vitro* (18–20) and increase muscle mass and strength *in vivo* (21). IOE showed no significant toxicity *in vivo* or animal models (22). However, no randomized, placebo-controlled trial in humans has explored the effects and safety of IOE in adults with low muscle mass. Based on previous studies, we hypothesized that IOE improves muscle strength and function in adults. Therefore, the present study investigates whether IOE increases muscle mass and strength with regular walking compared with placebo and normal walking in middle-aged and older adults with relatively lower muscle mass.

## Materials and methods

### Study design and ethical aspects

**Figure 1:**
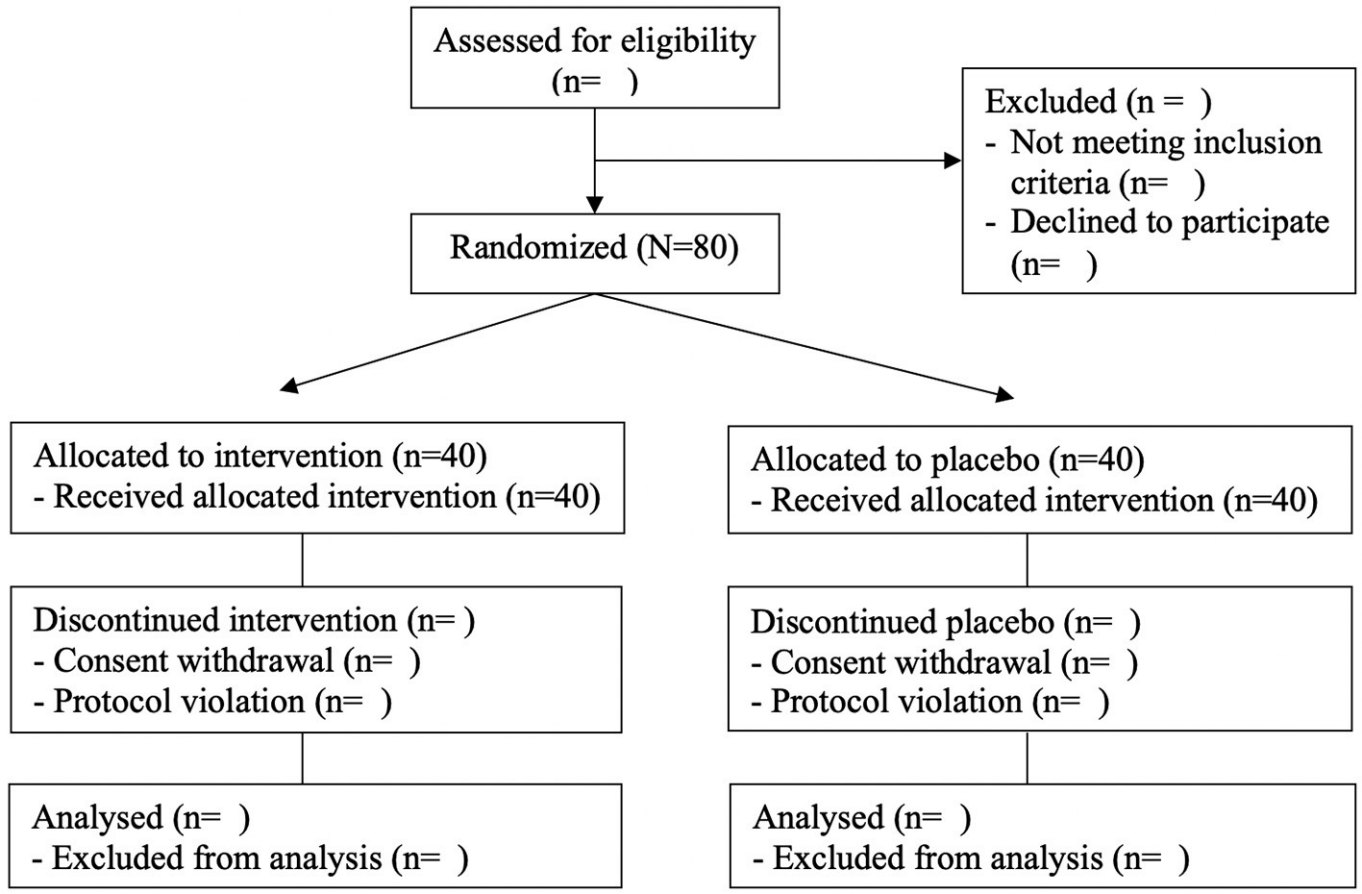
Tentative CONSORT flow diagram.

The study is designed as a randomized, placebo-controlled, double-blind clinical trial. All subjects gave their informed consent for inclusion before participating in the study. The study will be conducted per the Declaration of Helsinki, and the Institutional Review Board Ethics Committee at Pusan National University Yangsan Hospital (IRB No. 02-2020-029) approved the protocol. Table 1 shows the data collection schedule. The flow of participants through the controlled interventional trial is depicted in a tentative CONSORT conform diagram (Figure 1).

**Table 1:** Data collection schedule.

	**V1**	**V2**	**V3**	**V4**
**Participant information**				
Informed consent	**×**			
Selection (inclusion/exclusion criteria)	**×**	**×**		
Participant information	**×**			
History taking	**×**			
Physical examination	**×**	**×**	**×**	**×**
Vital sign	**×**	**×**	**×**	**×**
**Outcomes**				
Knee strength test		**×**		**×**
Hand grip strength test		**×**		**×**
Body composition analysis		**×**		**×**
Clinical laboratory test		**×**		**×**
**Questionnaires**				
Nutrition assessment		**×**		**×**
Quality of life		**×**		**×**
Waking log		**×**	**×**	**×**
**Test supplementation distribution**		**×**	**×**	
**Safety and compliance**				
Safety laboratory test	**×**			**×**
Adverse reaction			**×**	**×**
Concomitant drug taking	**×**	**×**	**×**	**×**
Compliance monitoring			**×**	**×**

V1, screening at visit 1; V2, baseline assessment at visit 2; V3, compliance and safety monitoring at visit 3; V4, end of intervention at visit 4.

### Participant recruitment and screening

Study candidates are recruited through bulletin boards and social media advertisements at a tertiary hospital in Yangsan-si, South Korea. Adults aged 50–85 are screened for eligibility according to the inclusion and exclusion criteria (Table 2). This study defined relatively low skeletal muscle mass as <110% of the standard lean mass measured using the body composition analyzer (InBody 720) (13). All subjects may withdraw their consent at any time during the study. Subjects of the following criteria will be considered to have dropped out or discontinued participation in the trial and will be excluded from the per-protocol analysis: failure to take the IOE supplementation or placebo for five consecutive days, failure to attend follow-up assessments, and poor compliance (< 80%). This study protocol is similar to our previously published study (13).

**Table 2:** Eligibility criteria.

**Inclusion criteria** •Aged between 50 and 85 years • Body mass index ranging from 18.5 to 30.0 kg/m^2^ • Relatively low skeletal muscle mass (< 110% of the standard lean mass measured using the body composition analyzer InBody 720) • Capable of normal physical activity
**Exclusion criteria** • Malignant tumor or severe cardiovascular diseases such as angina or myocardial infarction within the last 6 months • Any central bone fracture within the last 1 year • Uncontrolled hypertension (Blood pressure ≥ 160/100 mmHg) • Uncontrolled diabetes (fasting glucose level ≥ 160 mg/dL) • Uncontrolled hyperthyroidism or hypothyroidism • Abnormal liver function (aspartate aminotransferase or alanine aminotransferase concentration, greater than twice the upper limit of normal) • Abnormal renal function (creatinine concentration, greater than twice the upper limit of normal) • Severe gastrointestinal symptoms such as heartburn and indigestion • Those who participated in other drug clinical trials within the past 1 month • Psychiatric illnesses such as severe depression, schizophrenia, and drug addiction • Alcohol abusers • Those who are pregnant, lactating, or planning to become pregnant during the clinical trial period • Allergic reaction to the constituent foods • Those who are judged unsuitable by the researcher for other reasons

### Randomization

The procedure of randomization has been reported elsewhere (13). Eighty participants will be finally enrolled after undergoing baseline measurements. They are randomly assigned to either one of the intervention groups with IOE (IOE) or the control group with placebo through block randomization using randomized numbers and given identification numbers on recruitment. Randomization codes were created by an expert in statistics using nQuery Advisor 7.0. Those responsible for deciding on study eligibility and conducting the measurements are unaware of the randomization results throughout the whole study process.

### Study intervention

In a previous animal study (22), efficacy started to show from a 50 mg/kg dose of IOE, and IOE significantly upregulated the grip strength in a dose-dependent manner. However, both the administration of 100 and 200 mg/kg doses of IOE similarly fully recovered lean mass. Therefore, 100 mg/kg was selected as the efficacious dose of IOE for improving muscle strength for safety and efficacy in the present study. The dose was converted to a human equivalent dose based on the person's body surface area, 480 mg for individuals weighing 60 kg (23). Thus, 500 mg/60 kg was selected as the final dose. This dose of IOE satisfied all standards for hazardous substances in the preclinical toxicity test (22). IOE and placebo were supplied by ShinWoo Co., Ltd., Anyang-si, South Korea.

Participants are randomly assigned to the IOE group or placebo group. The IOE group (*n* = 40) will be administered 500 mg IOE supplement/day orally, that is, one 250 mg capsule 30 min after breakfast and after dinner, for 12 weeks. The placebo group (*n* = 40) will be administered identically the same quantity of the placebo. The placebo was identical in appearance to the IOE capsule but was filled with crystalline cellulose. The participants will be requested to log supplement intake in a diary whenever they take it, which was turned in along with the bottle to the researcher at every visit. Compliance will be assessed by counting the remaining capsules at every visit, as in our previous study (13).

### Measurements of outcomes and safety

As this is one of a series of studies verifying the benefits of foods for muscle, selecting and measuring optimal outcomes and safety were based on our previous studies (13, 23).

### Outcomes

All outcomes were measured at baseline and after 12 weeks. The primary outcome measure was the change from the baseline in the peak torque (TQ) at 60°/s of knee extension/flexion representing muscle function at 12 weeks of IOE or placebo use. The secondary outcome measures were changes in appendicular skeletal mass (ASM)/height^2^ (kg/m^2^) (appendicular skeletal mass index, ASMI), ASM/weight x 100 (skeletal muscle mass index, SMI), total body fat (%), trunk body fat (%), hand grip (kg), serum creatinine, pyruvate, lactate, and high sensitivity C-reactive protein during the 12-week treatment period.

### Muscle strength

Measurements of knee extension strength representing muscle strength and handgrip strength were conducted using the Biodex System 3 Pro isokinetic dynamometer (Biodex, Inc.) and a Jamar hydraulic hand dynamometer (Performance Health, Warrenville, IL, USA), respectively. The detailed measurement was described in our previous study (13).

### Body composition

Body mass index is calculated as weight (kg) divided by height in meters squared (m^2^). ASM is defined as the total lean soft-tissue mass of four limbs. A well-trained radiological technologist measured the body composition using dual-energy X-ray absorptiometry (Hologic Horizon W, Software: Apex Versions 5.6.0.5, Hologic Inc., Marlborough, MA). The detailed measurement was described in our previous study (13).

### Biochemical measurements for outcomes

All laboratory analyses will be performed in a central laboratory. After a 12-h overnight fast, blood samples are collected at baseline and at 12 weeks to evaluate the biomarkers of muscle metabolism of IOE. The serum creatinine will be measured using modified Jaffe's kinetic alkaline picrate method. Pyruvate will be measured by the enzymatic method, and hs-CRP will be measured by latex particle-enhanced central immunoturbidimetric assay on the AU5800 chemistry analyzer (Beckman Coulter, Brea, CA, USA). An ion-selective electrode assay will measure lactate concentrations using Stat Profile pHOx Ultra analyzer (Nova Biomedical, Waltham, MA, USA).

### Dietary intake and physical activities assessments

At the baseline and after 12 weeks of the trial, participants were asked to answer a questionnaire on dietary intake and physical activities that may influence muscle strength, mass, and metabolism changes. The detailed measurement is described in our previous study (13).

### Assessments of safety and additional benefits

Safety and additional benefits are assessed at each study visit based on adverse events (AEs), vital signs, physical examination, and laboratory test results (complete blood counts, liver enzymes, glucose, and creatinine) per protocol. Reports of any other adverse events or unpredicted allergic reactions are collected throughout the study. All AEs are coded using version 21.0 of the Medical Dictionary for Regulatory Activities. We previously described the biochemical and laboratory measurements in detail (24).

## Data analyses

### Sample size determination

MedCalc version 20.113 (MedCalc Software Ltd, Ostend, Belgium) was used to calculate the sample size, based on our previous study (13). The sample size was adjusted to 40 participants per group to allow for 20% dropouts. The detailed estimation is described in our previous study (13).

### Statistical analyses

Shapiro–Wilk's test will be used for the normality distribution assessment of all variables (Table 3). A *p* < 0.05 will be considered statistically significant. Data will be analyzed using IBM SPSS Statistics 22.0 (IBM Inc., Armonk, NY, USA) and R software version 3.6.2 (http://www.r-project.org/). Intention-to-treat analysis will be primary for comparisons of outcomes between the IOE and placebo groups, with multiple imputation of missing data. Five imputed data sets will be created for missing values at the 12-week follow-up for all variables. The outcome also will be compared between the two groups using a per-protocol (PP) analysis. Intergroup comparisons of baseline characteristics will be performed using the 2-sample *t*-test or Mann-Whitney's *U*-test for continuous variables and the chi-square test or Fisher's exact test for categorical variables. ANCOVA or rank ANCOVA will be used for the primary analysis, with adjustments for the variables if there is a difference between the two groups at the start of the study and there is a difference in exercise and diet during the study period, all of which will be calculated as a delta. All randomized patients exposed to at least one dose of study intervention will be included for safety analyses, as our previous study (13).

**Table 3:** Study variables.

	**Variables**
Primary outcome	Peak TQ at 60°/s of knee extension/flexion (right/left)
Secondary outcomes	ASM/height^2^ (ASMI), ASM/weight x 100 (SMI), Total body fat (%), Trunk body fat (%), Hand grip strength (kg), Creatinine, Pyruvate, Lactate, and high sensitivity C-reactive protein
	**Covariables**
Demography	Age, Gender, Height (cm), Weight (kg), Body mass index (kg/m^2^), Alcohol consumption (per week, amount of alcohol at once), Smoking status (pack, years), Co-morbidities
Vital sign	Blood pressure (mmHg), Heart rate (rpm)
Additional chemistry	Free fatty acid, Creatinin kinase, Tumor necrosis factor-α, interleukin-6, Malondialdehyde, HOMA-IR
Self-Administered Questionnaires	Nutrition assessment (24-h recall method), Quality of life (EQ-5D-3L, VAS), Physical activity (IPAQ), waking log

TQ, torque; ASM, appendicular skeletal mass; ASMI, appendicular skeletal mass index; SMI, skeletal muscle mass index; HOMA-IR, Homeostatic Model Assessment for Insulin Resistance; EQ-5D-3L, Euro-QoL-5 dimensions-3 levels; VAS, visual analgue scale; IPAQ, International Physical Activity Questionnaire.

## Discussion

Muscle loss is a lifelong process that begins around the age of 30. During this process, the amount of muscle tissue and the number and size of muscle fibers gradually decrease. Sarcopenia refers to decreased skeletal muscle, mainly distributed in the extremities. The result of sarcopenia is a gradual loss of muscle mass and strength (1). Sarcopenia can make you vulnerable to arthritis or falls and increase your risk of senility, disability, fractures, and death (5–7). The leading causes of sarcopenia include inadequate nutrition, impaired muscle adaptation to nutrition, mitochondrial dysfunction, oxidative stress, excessive mitochondrial autophagy, changes in hormone production and sensitivity, decreased neuroendocrine regulation, chronic inflammation, and decreased physical activity (2–4). A balanced intake of amino acids-rich milk, eggs, meat, and fish, flexibility and balance, resistance strength training, and endurance training help prevent muscle loss. In addition, various nutrient supplements as an available alternative are being tried to verify their effectiveness on muscle mass, muscle strength, and even physical function in adults with sarcopenia through preclinical and clinical trials.

The main active components of IOE are polyphenols-ishophloroglucin A (IPA) and diphlorethohydroxycarmalol (DPHC) (25). Previous studies revealed that IOE has anti-obesity and anti-diabetic properties (16–18). Although the exact mechanism IOE prevents sarcopenia has not been elucidated, several attempts have been made to clarify the mechanism (18–20). Various genes activate the catabolic pathway of skeletal muscle as age (25). According to studies, phosphatidylinositol 3-kinase (PI3K)-p85α and protein kinase B (Akt), which are involved in muscle proliferation, are down-regulated in sarcopenia with age (26, 27). Recently, systematic preclinical studies have been conducted to verify the effects of IOE or its active components on muscle health (17–22). Jayawardena et al. (22) found that IOE inhibited the adverse effect of lipid-impaired skeletal myogenesis *via* reversion of downregulation of phosphorylation of PI3K/Akt/mammalian target of rapamycin axis, which is responsible for protein synthesis. Yang et al. (28) reported that IPA and IO-derived IPA increased leg muscle width and grip strength in the DEX-induced muscle atrophy model compared with saline. In addition, PI3K mRNA levels were elevated in the soleus muscle tissues in the IPA group. Ryu et al. (21) found that IOE and its active component DPHC improved grip strength and ladder climbing responses and preserved lean mass of calf muscle atrophy induced by dexamethasone (DEX) *in vivo*. Also, treatment with IOE or DPHC restored the DEX-mediated reductions in PI3K and Akt mRNA levels in the gastrocnemius muscle, which increased protein synthesis. PI3K/Akt signaling pathway is a crucial pathway regulating protein synthesis and muscle hypertrophy. Then, this effect was verified in an *in vivo* aging model. IOE or DPHC recovered lean mass, decreased calf circumference, and improved physical activity such as grip strength and ladder climbing in sarcopenic 14-month-old female C57BL/6J mice. IOE and DPHC showed high reversibility for aging parameters such as 17β-estradiol levels and senescence-associated secretory phenotypes levels in 14-month-old female mice. Furthermore, they improved the muscular regeneration-related gene expressions of PI3K/Akt in calf muscle (22).

Based on previous *in vitro* and animal studies, our study aimed to verify that IOE increases muscle mass and muscle strength in humans. We designed a randomized, placebo-controlled clinical trial to assess the effects of IOE on muscle strength, muscle mass, and muscle biomarkers in adults with relatively low muscle mass. IO has been used as food in some Asian regions (29–31). Although a diet containing seaweeds is not common in Western countries, after many benefits of seaweed are revealed, seaweed begins to consume raw materials for healthy food (32). Through this study, it may be possible to recommend using IOE as a nutrient therapy for patients with sarcopenia. However, this study has some limitations; our study focused on middle-aged and elderly Asian subjects and was conducted in a single center, so the effect of IOE on people of varying ages and ethnicities is still unknown. Secondary, a 24-h dietary recall self-questionnaire assesses nutritional intake: this information may not include the everyday diet of participants. Lastly, this is a 12-week follow-up study, so it could not determine the long-term effect of IOE. Despite these limitations, this study has considerable value for the following reason. To the best of our knowledge, the current study will be the first randomized, double-blind, placebo-controlled trial to examine the efficacy and tolerability of IOE supplementation in adults with relatively low muscle mass. The nutritional intake and physical activity that might influence muscle strength and mass will be considered as covariates for transparency of results. The results of this study will provide clinical evidence for sarcopenia patients with nutrient treatment.

## Ethics statement

The studies involving human participants were reviewed and approved by the Ethics Committee of the Institutional Review Board at Pusan National University Yangsan Hospital. The patients/participants provided their written informed consent to participate in this study.

## Author contributions

SYL contributed to the conceptualization of the study. SRL, YL, and SYL designed the methodology of the work, had an active role in the process of participant recruitment. SRL and SYL wrote the work's draft and reviewed the final document. All authors contributed to the article and approved the submitted version.

## Conflict of interest

The authors declare that the research was conducted in the absence of any commercial or financial relationships that could be construed as a potential conflict of interest.

## Publisher's note

All claims expressed in this article are solely those of the authors and do not necessarily represent those of their affiliated organizations, or those of the publisher, the editors and the reviewers. Any product that may be evaluated in this article, or claim that may be made by its manufacturer, is not guaranteed or endorsed by the publisher.
